# Supplementary material for: Characterization of the physical properties of electron-beam-irradiated white rice and starch during short-term storage
Source: PLoS One. 2019 Dec 17;14(12):e0226633. doi: 10.1371/journal.pone.0226633 (PMC6917276; doi:10.1371/journal.pone.0226633)
Supplement: S1 Table — Values are means ± SD of three determinations (n = 3). Numbers following the lowercased letters mean significant differences. Different numbers in a row or a column indicate significant differences at p < 0.05. (PDF) [file pone.0226633.s001.pdf]

| Storage temperature (℃) | dose（kGy） | Water content (%) | | | | | |
|---|---|---|---|---|---|---|---|
| | | 0 days | 15 days | 30 days | 45 days | 60 days | 75 days |
| 37 | 0 | 15.50±0.00a(1) | 12.84±0.08d(3) | 13.43±0.05b(2) | 13.06±0.00b(3) | 12.91±0.05b(3) | 10.68±0.21a(4) |
| | 2 | 15.01±0.03b(1) | 12.83±0.05d(4) | 13.06±0.01c(3,4) | 13.49±0.16a(2) | 13.20±0.00a(2,3) | 10.63±0.31a(5) |
| | 4 | 14.87±0.05cd(1) | 13.15±0.04c(2) | 13.09±0.03c(2) | 12.84±0.01c(3) | 12.96±0.03b(2,3) | 10.98±0.18a(4) |
| | 6 | 14.78±0.08d(1) | 13.66±0.01a(2) | 13.59±0.06a(2) | 13.01±0.01bc(3) | 12.76±0.00c(4) | 10.69±0.13a(5) |
| | 8 | 14.96±0.02bc(1) | 13.39±0.07b(2) | 13.34±0.08b(2) | 13.16±0.00b(2,3) | 12.96±0.02b(3) | 10.79±0.31a(4) |
| 25 | 0 | 15.50±0.00a(1) | 13.70±0.07a(2,3) | 13.52±0.02c(3) | 13.96±0.02bc(2) | 13.85±0.03d(2,3) | 11.67±0.32a(4) |
| | 2 | 15.01±0.03b(1) | 13.21±0.04b(4) | 13.89±0.03a(3) | 13.98±0.02bc(3) | 14.18±0.00a(2) | 12.04±0.17a(5) |
| | 4 | 14.87±0.05cd(1) | 12.53±0.04c(4) | 13.31±0.00d(3) | 14.28±0.01a(2) | 13.92±0.02c(2) | 11.75±0.47a(5) |
| | 6 | 14.78±0.08d(1) | 13.09±0.12b(4) | 13.61±0.03b(3) | 14.07±0.01b(2) | 14.02±0.00b(2) | 11.49±0.04a(5) |
| | 8 | 14.96±0.02bc(1) | 13.68±0.08a(2) | 13.47±0.05c(2) | 13.85±0.15c(2) | 13.94±0.04c(2) | 11.89±0.49a(3) |
