# Supplementary material for: Characterization of the physical properties of electron-beam-irradiated white rice and starch during short-term storage
Source: PLoS One. 2019 Dec 17;14(12):e0226633. doi: 10.1371/journal.pone.0226633 (PMC6917276; doi:10.1371/journal.pone.0226633)
Supplement: S2 Table — Values are means ± SD of three determinations (n = 3). Numbers following the lowercased letters mean significant differences. Different numbers in a row or a column indicate significant differences at p < 0.05. (PDF) [file pone.0226633.s002.pdf]

Water bath temperature, storage temperature (°C)	dose (kGy)	Swelling power (g/g)					
		0 days	15 days	30 days	45 days	60 days	75 days
90, 37	0	12.52±0.86a(1)	10.88±0.13a(2)	10.24±0.06a(2,3)	10.01±0.42a(2,3,4)	9.35±0.13a(3,4)	9.00±0.22a(4)
	2	12.32±0.92ab(1)	10.93±0.01a(2)	10.22±0.08a(2)	8.76±0.70b(3)	8.61±0.42a(3)	8.74±0.55a(3)
	4	11.52±0.26abc(1)	10.55±0.06b(1)	8.10±0.10b(2)	8.00±0.69bc(2)	8.06±0.84ab(2)	8.13±0.44ab(2)
	6	10.78±0.33bc(1)	9.02±0.20c(2)	8.77±0.79b(2)	8.72±0.11b(2)	8.34±0.79a(2,3)	7.29±0.29bc(3)
	8	10.01±0.23c(1)	8.75±0.08c(2)	7.90±0.59b(2,3)	7.15±0.08c(3,4)	6.60±0.58b(4)	6.73±0.56c(4)
90, 25	0	12.52±0.86a(1)	10.45±0.00b(2)	10.16±0.05a(2,3)	9.60±0.10ab(2,3)	9.00±0.91a(3)	9.25±0.47a(2,3)
	2	12.32±0.92ab(1)	10.81±0.01a(2)	10.18±0.19a(2,3)	9.84±0.80a(2,3)	8.90±0.45a(3)	8.87±0.22a(3)
	4	11.52±0.26abc(1)	9.97±0.15c(2)	9.04±0.10b(3)	8.80±0.07bc(3)	8.42±0.39a(3)	8.65±0.44a(3)
	6	10.78±0.33bc(1)	8.84±0.04d(2)	8.78±0.09b(2)	8.35±0.09c(2,3)	8.00±0.34a(3)	8.51±0.41a(2,3)
	8	10.01±0.23c(1)	8.36±0.03e(2,3)	8.90±0.19b(2)	8.80±0.16bc(2)	7.93±0.44a(3)	7.02±0.11b(4)
70, 37	0	10.45±0.38a(1)	10.48±0.04a(1)	9.37±0.33a(2)	9.23±0.16a(2)	9.23±0.19a(2)	9.01±0.61a(2)
	2	9.99±0.77ab(1)	8.98±0.23b(1,2)	8.25±0.13ab(2)	8.57±0.78a(2)	7.82±0.50b(2)	8.28±0.22ab(2)
	4	9.23±0.04ab(1,2)	9.86±0.76ab(1)	8.23±0.57ab(2)	8.05±0.28a(2)	8.01±0.59b(2)	8.09±0.18ab(2)
	6	9.80±0.78ab(1)	9.61±0.33ab(1)	8.00±0.57b(2)	8.57±0.51a(1,2)	7.68±0.11b(2)	7.79±0.56ab(2)
	8	8.89±0.51b(1,2)	9.23±0.16b(1)	7.63±0.54b(3)	8.05±0.28a(2,3)	7.39±0.07b(3)	7.24±0.56b(3)
70, 25	0	10.45±0.38a(1)	9.74±0.05a(1,2)	8.13±0.64a(3)	8.01±0.27ab(3)	8.82±0.68a(2,3)	8.03±0.06a(3)
	2	9.99±0.77ab(1)	9.61±0.39a(1,2)	8.31±0.02a(2)	8.35±0.52a(2)	8.57±0.67a(2)	8.31±0.57a(2)
	4	9.23±0.04ab(1,2)	9.94±0.54a(1)	8.73±0.55a(2,3)	7.95±0.34ab(3)	8.83±0.51a(2,3)	8.42±0.42a(2,3)
	6	9.80±0.78ab(1)	9.70±0.49a(1)	8.30±0.22a(2)	8.17±0.26ab(2)	8.40±0.77a(2)	8.31±0.32a(2)
	8	8.89±0.51b(1)	8.30±0.23b(1,2)	7.91±0.58a(1,2)	7.34±0.29b(2)	7.77±0.25a(2)	7.78±0.52a(2)
