# Supplementary material for: Characterization of the physical properties of electron-beam-irradiated white rice and starch during short-term storage
Source: PLoS One. 2019 Dec 17;14(12):e0226633. doi: 10.1371/journal.pone.0226633 (PMC6917276; doi:10.1371/journal.pone.0226633)
Supplement: S3 Table — Values are means ± SD of three determinations (n = 3). Numbers following the lowercased letters mean significant differences. Different numbers in a row or a column indicate significant differences at p < 0.05. (PDF) [file pone.0226633.s003.pdf]

b*/L*, storage temperature (℃)	dose (kGy)	Swelling power (g/g)					
		0 days	15 days	30 days	45 days	60 days	75 days
b*, 37	0	6.72±0.18e(2)	6.65±0.15d(2)	6.29±0.06e(3)	6.96±0.15e(1)	6.07±0.15e(3)	7.01±0.24e(1)
	2	6.98±0.13d(4)	6.43±0.07d(5)	7.78±0.27d(2)	8.16±0.09d(1)	8.07±0.12d(1)	7.53±0.03d(3)
	4	7.75±0.20c(4)	9.23±0.24c(3)	9.98±0.17c(1,2)	10.20±0.17c(1)	10.00±0.27c(1,2)	9.84±0.09c(2)
	6	9.11±0.18b(4)	11.12±0.25b(3)	12.28±0.22b(2)	13.08±0.25b(1)	12.81±0.27b(1)	12.18±0.12b(2)
	8	10.01±0.06a(5)	12.34±0.26a(4)	13.94±0.61a(2)	14.52±0.33a(1)	13.79±0.13a(2)	13.06±0.18a(3)
b*, 25	0	6.72±0.18e(1)	6.36±0.25e(2,3)	6.67±0.28e(1)	6.73±0.11e(1)	6.55±0.18e(1,2)	6.23±0.10e(3)
	2	6.98±0.13d(3)	7.11±0.29d(2,3)	7.55±0.17d(1)	7.34±0.23d(1,2)	7.48±0.20d(1)	7.11±0.03d(2,3)
	4	7.75±0.20c(3)	7.72±0.43c(3)	8.84±0.08c(2)	9.29±0.36c(1)	9.24±0.15c(1)	8.58±0.05c(2)
	6	9.11±0.18b(4)	9.04±0.13b(4)	10.76±0.50b(2)	11.23±0.11b(1)	11.24±0.25b(1)	10.25±0.25b(3)
	8	10.01±0.06a(4)	10.38±0.12a(4)	12.71±0.16a(1)	12.83±0.43a(1)	11.99±0.30a(2)	11.07±0.27a(3)
L*, 37	0	96.00±0.20a(1)	95.12±0.37a(2)	94.17±0.20a(3)	94.25±0.24a(3)	93.01±0.15a(4)	95.16±0.24a(2)
	2	95.80±0.35a(1)	93.88±0.17b(3)	93.86±0.07ab(3)	93.24±0.30b(4)	93.28±0.19a(4)	94.85±0.37a(2)
	4	95.00±0.37b(1)	93.70±0.43b(3)	93.42±0.13b(3,4)	92.88±0.31b(5)	93.06±0.19a(4,5)	94.30±0.22b(2)
	6	94.80±0.33b(1)	92.97±0.12c(2)	92.01±0.16c(3)	91.74±0.23c(3)	92.00±0.28b(3)	92.09±0.21d(3)
	8	94.80±0.40b(1)	92.29±0.28d(2)	91.08±0.68d(3)	90.96±0.15d(3)	91.31±0.29c(3)	92.62±0.36c(2)
L*, 25	0	96.00±0.20a(1)	94.25±0.74a(3)	93.47±0.10ab(4)	94.09±0.14a(3)	95.03±0.22a(2)	95.34±0.19a(2)
	2	95.80±0.35a(1)	94.29±0.76a(2,3)	93.74±0.22a(3,4)	93.47±0.43b(4)	94.21±0.22b(3)	94.88±0.09b(2)
	4	95.00±0.37b(1)	94.21±0.42a(2,3)	93.32±0.41ab(4)	93.07±0.27b(4)	93.93±0.51b(3)	94.53±0.06c(1,2)
	6	94.80±0.33b(1)	93.28±0.14b(3)	93.02±0.50b(3)	92.26±0.20c(4)	93.06±0.24c(3)	94.16±0.27d(2)
	8	94.80±0.40b(1)	93.43±0.34ab(3)	91.86±0.31c(5)	91.97±0.41c(5)	92.88±0.29c(4)	94.12±0.26d(2)
