# Supplementary material for: Characterization of the physical properties of electron-beam-irradiated white rice and starch during short-term storage
Source: PLoS One. 2019 Dec 17;14(12):e0226633. doi: 10.1371/journal.pone.0226633 (PMC6917276; doi:10.1371/journal.pone.0226633)
Supplement: S4 Table — Values are means ± SD of three determinations (n = 3). Different letters in a column indicate significant differences at p < 0.05. (PDF) [file pone.0226633.s004.pdf]

Dose	Hardness		Stickiness		Elasticity		Appearance		Taste		Overall Acceptability	
(kGy)	25 ℃	37 ℃	25℃	37℃	25℃	37℃	25℃	37℃	25℃	37℃	25℃	37℃
0	5.29±0.09a	8.39±0.27a	0.44±0.01c	0.49±0.01b	0.75±0.02a	0.80±0.02a	6.85±0.07a	5.60±0.28a	7.05±0.35a	6.05±0.50a	73.20±0.42a	65.06±0.57a
2	4.62±0.08b	6.43±0.36b	0.67±0.01a	0.62±0.02a	0.69±0.02b	0.75±0.02a	6.75±0.07a	5.55±0.21a	7.15±0.50a	6.20±0.42a	75.60±0.99a	67.70±0.99a
4	3.10±0.10c	3.94±0.36c	0.49±0.02b	0.50±0.03b	0.63±0.03c	0.68±0.02b	4.60±0.28b	3.35±0.21b	5.10±0.42b	4.20±0.28b	64.20±1.70b	57.00±1.41b
6	2.74±0.18c	2.89±0.29d	0.41±0.02c	0.38±0.02c	0.62±0.01c	0.65±0.02b	2.80±0.14c	1.85±0.21c	3.70±0.28c	3.05±0.21c	55.65±0.92c	51.30±1.27c
8	2.90±0.17c	3.27±0.33cd	0.42±0.01c	0.51±0.02b	0.6±0.01c	0.69±0.02b	1.95±0.07d	0.75±0.07d	3.05±0.21c	2.35±0.21c	51.20±1.70d	41.95±2.76d
