# Supplementary material for: Characterization of the physical properties of electron-beam-irradiated white rice and starch during short-term storage
Source: PLoS One. 2019 Dec 17;14(12):e0226633. doi: 10.1371/journal.pone.0226633 (PMC6917276; doi:10.1371/journal.pone.0226633)
Supplement: Attachment — Submitted filename: Comments to Authors.docx [file pone.0226633.s005.docx]

PONE-D-19-15367

Comments to Authors-

-The authors extensively investigate the effect of electronic-beam irradiation on the storage quality of white rice at two different ambient temperatures. In general, the data collection is complete. However, there are some issues in the data presentation, result demonstration and discussion as well. Detailed comments are as follows:

**Introduction-**

-The difference between rice flour and starch should be better described in the introduction section. Indeed, this should be closely related to the results/discussion part where the effects of EBI on the rice quality properties are presented in Table1-4.

**Materials and methods-**

-L90-91 it is not clear that where these nutrient data come from. This should be better explained.

-L95- ‘a dose rate of 2kGy/h’ needs to be deleted because multiple doses of irradiation were applied, as stated in L96-97. Moreover, the duration of the irradiation treatment (which I think is critical) should be mentioned.

-L98- it was not described how the moisture content (at wet basis) was measured in the method section, and what the changes of this parameter would affect other properties such as Sp, rice color and storage time. In addition, the moisture content should also be included in the correlation analysis.

**Results and discussion-**

- Discussion is not sufficient (for example, L165-167, L216-218, L239-242, L284-285); it is suggested to refer to more relevant literatures and analyze each measure parameter on by one.

-The effect of EBI on the microbes should be mentioned and discussed somewhere as well.

**Tables and Figures -**

-In Table1-4, what do those numbers following the lower letters mean? It should be explained in the table footnote. In the footnote of all the four tables: ‘In a row, no numbers in common are significantly different (*p* < 0.05). In a column within one RVA pasting property, no letters in common are significantly different (*p* < 0.05)’. Consider revising - ‘different numbers in either a row or a column indicate significant differences at *p* < 0.05’. And the same footnote doesn’t need to be repeated four times.

-The font in Fig 1-3 looks small and unnormal. Consider revising.

- In Fig.3, the meaning of the letters/numbers above the bars should be explained in the figure legend.

Overall, the text should be carefully checked for English spelling and grammar (singular/plural, tenses, use of articles…); it is suggested to find a native English-speaking expert or a professional editing service to modify the whole manuscript. The literatures format should be carefully checked as well.
