# Supplementary material for: Characterization of the physical properties of electron-beam-irradiated white rice and starch during short-term storage
Source: PLoS One. 2019 Dec 17;14(12):e0226633. doi: 10.1371/journal.pone.0226633 (PMC6917276; doi:10.1371/journal.pone.0226633)
Supplement: Attachment — Submitted filename: Response to reviewers.docx [file pone.0226633.s006.docx]

We would like to express our sincere thanks to the reviewers for the constructive comments.

**Response to Reviewer #1**

**Comments 1: Introduction: Explain well the topic but it is concentrated on irradiation effect on microorganisms which is not treated in this work.**

**A section of studying the effect on microorganisms should be added in this manuscript.**

Response: Thank you for your kind suggestion. This work was based on the effect of electron-beam irradiation (EBI) on microorganisms. Thus, we discussed the topic in depth in the introduction. In our previous work entitled “Effect of electron beam irradiation on storability of brown rice and milled rice,” we found that the total viable bacterial count was significantly reduced from around 3×10^3^ CFU/g at 0 kGy to 10 and 0 CFU/g at 5 kGy. Therefore, the present work did not anymore focus on the effects of EBI on microorganisms. The explanation for this point has been added in the introduction **(seen in page 5, 6, line 86-90).** We hope that you will be satisfied with our revisions.

**Comments 2: Line 98-99 should be in the result**

Response: Thank you for this comment. The water content analysis was added in the results **(seen in page 11, line 167-177; Fig 1)**.

**Comments 3: Line 96-97: you have to explain the choice of the dose spectrum Irradiation, starch isolation, pasting proprieties: lack of reference**

Response: The explanation on the choice of the dose spectrum irradiation **(seen in page 6, line 101)** and references of starch isolation **(seen in page 7, line 112-113)** and pasting properties **(seen in page 8, line 126-127)** has been added in the manuscript.

**Comments 4: Figures are not clear**

Response: The figures have been modified **(seen in Figs 1-4)**. Thank you very much.

**Response to Reviewer #2**

**Comments 1: Introduction-**

**-The difference between rice flour and starch should be better described in the introduction section. Indeed, this should be closely related to the results/discussion part where the effects of EBI on the rice quality properties are presented in Table1-4.**

Response: Thank you for your suggestion. The difference between rice flour and starch has been discussed in the introduction **(seen in page 5, line 79-85)**, and the pasting properties related to it have also been presented **(seen in page 19, 20, line 237-247)**.

**Comments 2: Materials and methods-**

**-L90-91 it is not clear that where these nutrient data come from. This should be better explained.**

Response: Thank you for highlighting this point. The determination methods and references of nutrient data have been added in the manuscript **(seen in page 7, line 107-109)**.

**Comments 3: -L95-** **‘a dose rate of 2kGy/h’ needs to be deleted because multiple doses of irradiation were applied, as stated in L96-97. Moreover, the duration of the irradiation treatment (which I think is critical) should be mentioned.**

Response: The part “a dose rate of 2 kGy/h” has been deleted **(seen in page 6, line 100)**. Moreover, the irradiation duration was fixed because the item was placed on a speed-constant conveyor belt and passed through fixed distance. The variance during electron beam irradiation was irradiation intensity controlled by electronic speed flow that varied with the irradiation dose. The 5 s duration of irradiation has been added to the manuscript following your suggestion **(seen in page 6, line 99)**.

**Comments 4: -L98- it was not described how the moisture content (at wet basis) was measured in the method section, and what the changes of this parameter would affect other properties such as Sp, rice color and storage time. In addition, the** **moisture content should also be included in the correlation analysis.**

Response: The methods of determining the moisture content of white rice and the corresponding results (including the figure legend) have been added in the manuscript **(seen in page 7, line 107-108; page 11, line 167-177; and Fig 1)**. The moisture content has also been included in the correlation analysis **(seen in Tables 5-6)**. In accordance with the correlation analysis, water content has a significant correlation with storage time and swelling power **(seen in page 23, line 300-301; page 24, line 317-320)**. Therefore, the relationship between water and Sp was discussed **(seen in page 21, line 262-267)**.

**Comments 5: Results and discussion-**

**- Discussion is not sufficient (for example, L165-167, L216-218,** **L239-242, L284-285); it is suggested to refer to more relevant literatures and analyze each measure parameter on by one.**

Response: Thank you for your suggestion. We have analyzed each parameter individually, which we hope will now meet your standard **(seen in page 12, line 186-191; seen in page 13, line 203-208; seen in page 20, line 254-259; seen in page 23, 24, line 307-317)**.

**Comments 6: The effect of EBI on the microbes should be mentioned and discussed somewhere as well.**

Response: Thank you for your kind suggestion. In our previous work entitled “Effect of electron beam irradiation on storability of brown rice and milled rice,” we found that total viable bacterial count was significantly reduced from approximately 3×10^3^ CFU/g at 0 kGy to 10 and 0 CFU/g at 5 kGy **(seen in page 5, 6, line 86-90)**. Therefore, the present work did not anymore focus on the effects of EBI on microorganisms. A relevant explanation has been added in the introduction.

**Comments 7: Tables and Figures -**

**-In Table1-4, what do those numbers following the lower letters mean? It should be explained in the table footnote. In the footnote of all the four tables: ‘In a row, no numbers in common are significantly different (p < 0.05). In a column within one RVA pasting property, no letters in common are significantly different (p < 0.05)’. Consider revising - ‘different numbers in either a row or a column indicate significant differences at p < 0.05’. And the same footnote doesn’t need to be repeated four times.**

Response: We appreciate your helpful suggestions. The numbers that follow the lowercased letters indicate significant differences. This point has been explained in the table footnote. The footnote was as follows: “In a row, no numbers in common are significantly different (p < 0.05). In a column within one RVA pasting property, no letters in common are significantly different (p < 0.05)” has been revised to “Different numbers in a row or a column indicate significant differences at p < 0.05.” The repetitive footnotes have been deleted **(seen in page 18, line 224-225)**.

**Comments 8: -The font in Fig 1-3 looks small and unnormal. Consider revising.**

Response: We appreciate this comment. The font in Figs. 1–3 has been revised **(seen in Figs. 2–4)**.

**Comments 9: - In Fig.3, the meaning of the letters/numbers above the bars should be explained in the figure legend.**

Response: Thank you for this reminder. The meaning of the letters above the bars has been explained in the figure legend **(seen in page 28, line 356)**.

**Comments 10: Overall, the text should be carefully checked for English spelling and grammar (singular/plural, tenses, use of articles…); it is suggested to find a native English-speaking expert or a professional editing service to modify the whole manuscript. The literatures format should be carefully checked as well.**

Response: We appreciate your helpful suggestions. The entire manuscript has been rechecked by a professional editing service. The literature format has been carefully checked. We hope that the revised version would meet the standard of PLOS ONE.

We are grateful for your helpful comments.

**References:**

1.	Goel PK, Singhal RS, Kulkarni PR. Studies on interactions of corn starch with casein and casein hydrolysates. Food Chemistry. 1999; 64(3): 383-9. doi: 10.1016/s0308-8146(98)00134-4. PubMed PMID: WOS: 000077881500014.

2.	Luo XH, Li YL, Yang D, Xing JL, Li K, Yang M, et al. Effects of electron beam irradiation on storability of brown and milled rice. Journal of Stored Products Research. 2019; 81: 22-30. doi: 10.1016/j.jspr.2018.12.003. PubMed PMID: WOS: 000463305500004.

3.	Chen X, Jin Y, Meng Y, Xie J, Liu C. Effect of high-energy electron beam irradiation on eating quality of rice. Food Science. 2016; 37(3): 71-4. PubMed PMID: CSCD: 5638309.

4.	Zhong F, Li Y, Ibanz AM, Oh MH, McKenzie KS, Shoemaker C. The effect of rice variety and starch isolation method on the pasting and rheological properties of rice starch pastes. Food Hydrocolloids. 2009; 23(2): 406-14. doi: 10.1016/j.foodhyd.2008.02.003. PubMed PMID: WOS: 000260283900019.

5.	Du SK, Jiang HX, Ai YF, Jane JL. Physicochemical properties and digestibility of common bean (Phaseolus vulgaris L.) starches. Carbohydrate Polymers. 2014; 108: 200-5. doi: 10.1016/j.carbpol.2014.03.004. PubMed PMID: WOS: 000336193600026.

6.	Rani MRS, Bhattacharya KR. Rheology of rice-flour pastes: Relationship of paste breakdown to rice quality, and a simplified Brabender viscograph test. Journal of Texture Studies. 1995; 26(5): 587-98. doi: 10.1111/j.1745-4603.1995.tb00806.x. PubMed PMID: WOS: A1995TR67700008.

7.	Vandeputte GE, Vermeylen R, Geeroms J, Delcour JA. Rice starches. III. Structural aspects provide insight in amylopectin retrogradation properties and gel texture. Journal of Cereal Science. 2003; 38(1): 61-8. doi: 10.1016/s0733-5210(02)00142-x. PubMed PMID: WOS: 000183868800006.

8.	Bashir K, Swer TL, Prakash KS, Aggarwal M. Physico-chemical and functional properties of gamma irradiated whole wheat flour and starch. Lwt-Food Science and Technology. 2017; 76: 131-9. doi: 10.1016/j.lwt.2016.10.050. PubMed PMID: WOS: 000390726400017.

9.	Ai Y, Jane JL. Gelatinization and rheological properties of starch. Starch-Starke. 2015; 67(3-4): 213-24. doi: 10.1002/star.201400201. PubMed PMID: WOS: 000350977700001.

10.	Beleia A, Miller RA, Hoseney RC. Starch gelatinization in sugar solutions. Starch-Starke. 1996; 48(7-8): 259-62. doi: 10.1002/star.19960480705. PubMed PMID: WOS: A1996VH67900004.

11.	Kohyama K, Nishinari K. Effect of soluble sugars on gelatinization and retrogradation of sweet-patato starch. Journal of Agricultural and Food Chemistry. 1991; 39(8): 1406-10. doi: 10.1021/jf00008a010. PubMed PMID: WOS: A1991GC08700010.

12.	Sasaki T, Matsuki J. Effect of wheat starch structure on swelling power. Cereal Chemistry. 1998; 75(4): 525-9. doi: 10.1094/cchem.1998.75.4.525. PubMed PMID: WOS: 000074899600024.

13.	Sung WC. Effect of gamma irradiation on rice and its food products. Radiation Physics and Chemistry. 2005; 73(4): 224-8. doi: 10.1016/j.radphyschem.2004.08.008. PubMed PMID: WOS: 000229717200006.

14.	Martin M, Fitzgerald MA. Proteins in rice grains influence cooking properties. Journal of Cereal Science. 2002; 36(3): 285-94. doi: 10.1006/jcrs.2001.0465. PubMed PMID: WOS: 000178869300002.
