# Supplementary material for: Characterization of the physical properties of electron-beam-irradiated white rice and starch during short-term storage
Source: PLoS One. 2019 Dec 17;14(12):e0226633. doi: 10.1371/journal.pone.0226633 (PMC6917276; doi:10.1371/journal.pone.0226633)
Supplement: Attachment — Submitted filename: Response to reviewers.docx [file pone.0226633.s007.docx]

We would like to express our sincere thanks to your comments.

**Response to Editor Comments**

**Comments 1: The abstract should be improved an introductive sentence should be added.**

Response: An introductive sentence has been added in manuscript. **(seen in page 2, line 20-21)**

**Comments 2: Authors need to add references to the text in introduction section. (ex. Line 35 to 40).**

Response: References to the text in introduction section (ex. Line 35 to 40) have been added. **(seen in page 3, line 38-41)**

**Comments 3: The whole manuscript should be revised for some syntax and grammar errors before final acceptance.**

Response: We appreciate your kind suggestions. The entire manuscript has been rechecked by a professional editing service. We hope that the revised version would meet the standard of PLOS ONE.

**References:**

1.	FAO. Database: figshare [Internet]. Available from: http://www.fao.org/faostat/en/#data/QC.

2.	Zhou Z, Wang X, Si X, Blanchard C, Strappe P. The ageing mechanism of stored rice: A concept model from the past to the present. Journal of Stored Products Research. 2015; 64: 80-7. doi: 10.1016/j.jspr.2015.09.004. PubMed PMID: WOS:000366780900013.

3.	Yadav DN, Anand T, Sharma M, Gupta RK. Microwave technology for disinfestation of cereals and pulses: An overview. Journal of Food Science and Technology-Mysore. 2014; 51(12): 3568-76. doi: 10.1007/s13197-012-0912-8. PubMed PMID: WOS:000345916400004.

4.	Gregory AG, Toshitaka U, Fumihiko T, Daisuke H. Effect of vapors from fractionated samples of propolis on microbial and oxidation damage of rice during storage. Journal of Food Engineering. 2008; 88(3): 341-52. doi: 10.1016/j.jfoodeng.2008.02.019. PubMed PMID: WOS: 000256815400007
