# Supplementary material for: Characterization of the physical properties of electron-beam-irradiated white rice and starch during short-term storage
Source: PLoS One. 2019 Dec 17;14(12):e0226633. doi: 10.1371/journal.pone.0226633 (PMC6917276; doi:10.1371/journal.pone.0226633)
Supplement: Attachment — Submitted filename: Response to reviewers.docx [file pone.0226633.s008.docx]

**Response to Editor Comments**

**Comments 1:** **The authors indicated in the text that there is a significant variation in different parameters however no statistical analysis is done to confirm. Please add ANOVA to figures (figures 1 to figures 4) and specifies if the comparison is made between groups with different irradiation doses or different storage time (days).**

Response: Thank you for your helpful comments. **a)** We have made some mistakes in using “significant” and “significantly”, and we have revised the content in the manuscript to make the statement more rigorous **(seen in page 11, line 169-170 and 180; page 20, line 250-252 and line 257-258; page 21, line 272-273; page 22, line 285-286; page 27, line 338-340; page 35 and 36, line 480-493)**. **b)** The ANOVA has been added in Tables S1-4 in supporting files. **c)** we have revised figure 4 to make it more reasonable and have rechecked the font in other three figures.
